# The role of the tumor microenvironment in endocrine therapy resistance in hormone receptor-positive breast cancer

**DOI:** 10.3389/fendo.2023.1261283

**Published:** 2023-10-13

**Authors:** Jie Yuan, Li Yang, Zhi Li, Hua Zhang, Qun Wang, Jun Huang, Bei Wang, Chakrabhavi Dhananjaya Mohan, Gautam Sethi, Geng Wang

**Affiliations:** ^1^ Department of Endocrine and Vascular Surgery, Taihe Hospital, Hubei University of Medicine, Hubei, China; ^2^ Department of Pharmacology, Yong Loo Lin School of Medicine, National University of Singapore, Singapore, Singapore; ^3^ Department of Clinical Laboratory Medicine, Taihe Hospital, Hubei University of Medicine, Hubei, China; ^4^ Department of Studies in Molecular Biology, University of Mysore, Manasagangotri, Mysore Karnataka, India; ^5^ FEST Division, CSIR-Indian Institute of Toxicology Research, Lucknow, Uttar Pradesh, India

**Keywords:** breast cancer, endocrine therapy resistance, estrogen, tumor microenvironment, drug resisitance

## Abstract

Endocrine therapy is the prominent strategy for the treatment of hormone-positive breast cancers. The emergence of resistance to endocrine therapy is a major health concern among hormone-positive breast cancer patients. Resistance to endocrine therapy demands the design of newer therapeutic strategies. The understanding of underlying molecular mechanisms of endocrine resistance, components of the tumor microenvironment (TME), and interaction of resistant breast cancer cells with the cellular/acellular components of the intratumoral environment are essential to formulate new therapeutic strategies for the treatment of endocrine therapy-resistant breast cancers. In the first half of the article, we have discussed the general mechanisms (including mutations in estrogen receptor gene, reregulated activation of signaling pathways, epigenetic changes, and cell cycle alteration) responsible for endocrine therapy resistance in hormone-positive breast cancers. In the latter half, we have emphasized the precise role of cellular (cancer-associated fibroblasts, immune cells, and cancer stem cells) and acellular components (collagen, fibronectin, and laminin) of TME in the development of endocrine resistance in hormone-positive breast cancers. In sum, the article provides an overview of the relationship between endocrine resistance and TME in hormone-positive breast cancers.

## 1 Introduction

Breast cancer is the most common malignant tumor in women, which seriously endangers women’s health. Hormone receptor-positive breast cancer accounts for approximately 60-75%, and endocrine therapy can significantly improve the prognosis of hormone receptor-positive breast cancer patients (1). Endocrine therapy drugs can be categorized as selective estrogen receptor modulators (SERM, e.g., tamoxifen), aromatase inhibitors (e.g., letrozole and exemestane), selective estrogen receptor degrader (SERD, e.g., fulvestrant), and progestin. Some patients do not respond to these treatments. Drug resistance can be of two types namely primary and secondary, in which the former population express hormone receptor, but is not sensitive to endocrine therapy whereas the latter population responds to endocrine therapy initially and acquire resistance gradually with continued endocrine therapy (2). The mechanisms of drug resistance in endocrine therapy are complicated, it is not caused by a single factor, but a combination of multiple factors, which is also the reason for the poor therapeutic effect of drugs developed for a single factor in clinical practice (3). Currently, the mechanisms of endocrine therapy resistance in breast cancer have been found to include estrogen receptor gene abnormalities (4), deregulated activation of cell signaling pathways (5), epigenetic regulation (6), alteration of cell cycle regulation (7), and the existence of tumor stem cell-like cells (8). In recent years, the tumor microenvironment (TME) has been found to be involved in the process of drug resistance in endocrine therapy of breast cancer and has become a current research hotspot in cancer therapeutics (9). TME refers to the local environment in which the tumor cells live along with various types of other cells in the presence of non-cellular components. There are complex bidirectional signaling and mutual influences between tumor cells and TME, which are closely related to the biological behavior of tumor cells, such as survival, metastasis, and drug resistance (10). Lymphocytes (T cells and B cells), dendritic cells, mast cells, tumor-associated macrophages, other immune cells, cancer-associated fibroblasts, adipocytes, endothelial cells and tumor stem cell-like cells, etc. are the cellular components of TME. Non-cellular fraction is contributed by tangible components and intangible components. Tangible components are constituents of extracellular matrix (ECM) such as collagen, fibronectin, laminin, aminoglycan and proteoglycan whereas intangible components include soluble factors such as growth factors, cytokines and chemokines. They also include some hormones, proteases (such as matrix metalloproteinases), and exosomes. This paper summarizes the mechanisms of endocrine therapy resistance in breast cancer with special emphasis on the role of TME.

## 2 General mechanisms of endocrine therapy resistance in breast cancer

### 2.1 Estrogen receptor gene abnormalities

Estrogen receptor (ER) is a member of the nuclear steroid receptor superfamily and the variants of ER are ERα and ERβ. Classically, the interaction of estrogen with ER in the nucleus results in its activation followed by transcription of its target genes. Whereas the ER located on the cell membrane cannot directly initiate gene transcription when it binds to estrogen. Instead, it activates other signaling pathways to promote ER-related gene transcription. ER is the target of endocrine therapy for breast cancer as the ER-driven genes have oncogenic functions. Endocrine therapy in breast cancer revolves around antagonizing the binding of estrogen and ER, degrading ER, and inhibiting the production of estrogen (11). Estrogen receptor alpha gene 1 (ESR1) encodes ER, and the DNA segment coding for the ligand binding domain of ER is the hotspot of mutation in ESR1. Studies have found that some mutations in this region result in the transcriptional function of ER in an estrogen-independent manner, leading to endocrine therapy resistance (12). The incidence of ESR1 mutation in primary breast cancer is less than 1%, while it is more than 20% in aromatase inhibitor-resistant breast cancer patients (4). In addition, the gene fusion of ESR1 is also associated with the resistance of breast cancer to endocrine therapy (13).

### 2.2 Deregulated activation of cellular pathways

#### 2.2.1 Growth factor receptor pathway

Growth factor receptors including epidermal growth factor receptor (EGFR), human epidermal growth factor receptor 2 (HER2), vascular endothelial growth factor receptor (VEGFR), insulin-like growth factor-1 receptor (IGF-1R), and fibroblast growth factor receptor (FGFR) exert are activated by interacting with a variety of growth factors, cytokines, hormones and other ligands. Some of these receptors stimulate the activation of PI3K/AKT/mTOR (Phosphatidylinositol 3-kinase/protein kinase B (PKB, also known as AKT)/mammalian target of rapamycin (mTOR), MAPK/ERK (mitogen-activated protein kinase/extracellular signal-regulated kinase) and other signaling pathways to phosphorylate and activate the transcriptional function of ER in an estrogen-independent manner contributing to resistance against endocrine therapy (14, 15).

#### 2.2.2 PI3K/AKT/mTOR pathway

PI3K/AKT/mTOR signaling pathway promotes the proliferation, differentiation and migration of tumor cells (16). PI3K/AKT/mTOR axis can be activated by either upstream receptor tyrosine kinases or mutations in genes coding for pathway proteins (17). Activated PI3K/AKT/mTOR signaling pathway promotes the phosphorylation of the N-terminal ligand-independent activation function domain of ER resulting in ligand-independent ER activation (18). Some clinical studies have indicated that Everolimus (a mTOR inhibitor) in combination with endocrine therapy extends progression-free survival in hormone receptor-positive breast cancer patients (19). About 40% of hormone receptor-positive breast cancer patients harbor mutations in PIK3CA and the combination of alpelisib-Fulvestrant treatment (Fulvestrant is an ER antagonist) extended the progression-free survival of hormone receptor-positive breast cancer patients harboring PIK3CA-mutation who had received endocrine therapy previously (20). In 2019, the FDA approved the use of alpelisib in combination with fulvestrant for hormone receptor-positive and HER2-negative advanced breast cancer patients harboring PIK3CA-mutation suggesting the importance of inhibition of PI3K/AKT/mTOR axis in hormone receptor-positive breast cancers (21).

#### 2.2.3 MAPK/ERK signaling pathway

The MAPK/ERK pathway is one of the most classical intracellular signaling pathways which is involved in tumor progression and drug resistance. The activation of the MAPK/ERK signaling axis phosphorylates ER, increases the sensitivity of ER to estrogen, and thereby contributes to endocrine therapy resistance (22). In addition, it communicates with other signaling pathways and cooperates to promote the proliferation and migration of tumor cells (23).

#### 2.2.4 Other signaling pathways

About 60 to 80% of breast cancer patients express androgen receptor (AR) and approximately 90% of ER-positive breast cancer patients express AR (24). Studies have shown that patients who are on adjuvant tamoxifen treatment with an AR : ERα ratio (≥ 2.0) had a beyond two-fold elevated risk of therapeutic failure concluding that AR : ERα ratio could be a predictor of the response to endocrine therapy (25). Studies have found that the expression of Wnt signaling pathway-related genes is increased in tamoxifen-resistant breast cancer cells, and inhibition of the Wnt pathway restores the sensitivity of breast cancer cells to tamoxifen (26). Other studies have confirmed that activation of the Wnt pathway is an early adaptive response to endocrine therapy in breast cancer. An early combination of endocrine therapy drugs with Wnt signaling pathway inhibitors reduces the emergence of endocrine therapy resistance (27).

### 2.3 Epigenetic regulation

Epigenetics refers to “the study of changes in gene function that are mitotically and/or meiotically heritable and that do not entail a change in DNA sequence” (28). Some chemical modifications either in the DNA (such as methylation of cytosine) or histones (such as acetylation, methylation, etc.) decide the fate of gene expression. Downregulation of ER expression is linked to resistance of breast cancer to endocrine therapy (11). The expression of ER can be reversibly and genetically reduced by epigenetic regulation, leading to endocrine therapy resistance (6). The hypermethylation of CpG islands catalyzed by DNA methyltransferase in the promoter region of the ESR1 gene inhibits the expression of ER. Histone deacetylases (HDACs) removed acetyl marks from the target histones which results in the condensation of chromatin and subsequent suppression of gene expression. A phase-II clinical trial performed on hormone therapy-resistant breast cancer patients demonstrated that a combination of vorinostat (an HDAC inhibitor) and tamoxifen displayed the reversal of endocrine resistance (29). The similar observations were also observed in cell-based studies (30). MicroRNAs (miRNAs) have also been studied in connection with epigenetics and endocrine resistance in breast cancer. miRNAs are a class of non-coding RNAs of approximately 22 nucleotides in length and are involved in the regulation of target gene expression (31, 32). As the commonest mechanism, miRNAs interact with the 3’untranslated region of their target mRNA and repress their translation by promoting their degradation. Zhao and colleagues demonstrated that miR-221/miR-222 and ERα have a reciprocal relationship concerning their expression. miR-221/miR-222 were found to directly interact with the 3’untranslated region of ERα mRNA. Ectopic expression of miR-221/miR-222 led to decline in the expression of ERα and showed tamoxifen resistance whereas the knockdown of miR-221/miR-222 displayed opposite effects (33, 34). A case control study also demonstrated the elevated expression of miR-221 of tamoxifen-resistant breast cancer patients (35).

### 2.4 Abnormal cell cycle regulation

The cell cycle is a highly coordinated event which drives the proliferation of cells with the involvement of a wide range of proteins including various cyclins and cyclin-dependent kinases (CDKs). The deregulated cell cycle is the hallmark event in cancers. The transition from G1 to S phase is the first step of the cell cycle in which cyclin D1 forms a complex with CDK4/6 to induce the phosphorylation of retinoblastoma protein with the subsequent release of transcription factor E2F. Overactivation of the cyclin D1/CDK4/6/RB/E2F regulatory axis leads to accelerated tumor cell proliferation and leads to drug resistance (36). Studies have indicated that ERα-driven E2F transcription has the ability to mediate resistance to estrogen deprivation (36). Inhibition of CDK4/6 significantly improves the progression-free survival and overall survival of breast cancer patients who are resistant to endocrine therapy. Currently, several CDK4/6 inhibitors, such as palbociclib, abemaciclib and ribociclib have been approved for use in combination with endocrine therapies in patients with ER-positive advanced breast cancer (37, 38).

## 3 The TME and endocrine therapy resistance

### 3.1 Cellular components include cancer-associated fibroblasts, several immune cells, adipocytes, endothelial cells and tumor stem cell-like cells, etc. They play significant role in endocrine therapy resistance of breast cancer.

#### 3.1.1 Cancer-associated fibroblasts

Cancer-associated fibroblasts (CAFs) are one of the major cellular components of tumor stroma. The decreased expression of caveolin-1 and increased expression of α-SMA and FAP make the CAFs different from normal fibroblasts (NFs) (39). The exact origin of CAFs is not fully understood. Studies have shown that CAFs can be derived from NFs, bone marrow-derived mesenchymal stem cells, endothelial cells or epithelial cells, and even tumor cells after EMT (40). In an interesting study, the role of CAF subsets on estrogen responsiveness to estrogen in ER-positive breast cancer cells was studied. In ER-positive breast cancer patient-derived tissue samples, two subsets of CAFs were identified in the TME based on the expression of CD146 and designated as CD146^+^ and CD145^-^ cells. In ER-positive breast cancer cells, CD145^-^ cells were found to reduce the expression of ER, as well as lessened the sensitivity of tumor cells to tamoxifen whereas CD145^+^ cells displayed opposite effects indicating that the composition of CAF-subsets in TME plays a key role in regulating the therapeutic response to endocrine therapy in estrogen-positive breast cancer patients (41). CAFs in breast cancer tissue express aromatase, which significantly increases the estrogen levels in TME than that of normal breast tissue and promotes the growth of tumor cells. Studies have reported that aromatase expression in breast cancer tissue is an independent risk factor for breast cancer prognosis (42). However, no study has confirmed the correlation between aromatase expression in tumor tissue and endocrine therapy resistance.

CAFs induce angiogenesis, ECM remodeling, and energy metabolism reprogramming by secreting growth factors, cytokines, and proteases, which further promote tumor cell growth, invasion, and metastasis (43, 44). Interleukin-6 (IL-6) and matrix metalloproteinases (MMPs) secreted by CAFs activate downstream signaling pathways such as PI3K/AKT/mTOR and MAPK/ERK signaling pathways, leading to endocrine therapy resistance. Inhibition of these signaling pathways significantly improves the progression-free survival of ER-positive patients with advanced breast cancer (45). Tamoxifen resistance was also found to be contributed by G-protein-coupled estrogen receptor 30 (GPR30) which is expressed in CAFs. GPR30 was found to be elevated in CAFs in tamoxifen-insensitive tumors compared to their sensitive counterpart. GPR30 triggered PI3K/mTOR axis to elevate the extracellular secretion of HMGB1 (High mobility group box 1) in CAFs which led to the activation of the MEK/ERK axis in neighboring ERα-positive breast cancer cells and thereby induction of autophagy to accelerate tamoxifen resistance (46).

In addition, CAFs remodel the ECM to form a barrier between tumor cells and immune cells, preventing immune cell infiltration and leading to immune escape (43). Fibronectin (FN), secreted by CAFs, is one of the major components of the ECM. FN in the ECM binds to β1 integrin on the tumor cell membrane, leading to the activation of downstream signaling pathways, which further promotes endocrine therapy resistance (47). Our previous study found that FN secreted by CAFs activates PI3K/AKT signaling pathway through β1 integrin on the surface of tumor cells, inducing EMT of breast cancer cells and contributing to endocrine therapy resistance (48).

In addition, CAFs provide metabolic raw materials for tumor cells, such as lactate, glutamine and pyruvate, which increase the mitochondrial function of breast cancer cells and reduce cell apoptosis (49). Besides, reactive oxygen species (ROS) produced by CAFs during energy metabolism induce EMT and stem cell characteristics of tumor cells through COX-2/NF-κB/HIF-1 signaling pathway, leading to tamoxifen resistance in breast cancer (50). Some studies have reported that CAFs-induced endocrine therapy resistance can be alleviated by mitochondrial toxic drugs such as metformin and arsenic trioxide, and relevant clinical trials are currently underway (51, 52).

#### 3.1.2 Immune cells

Immune cells in the tumor stroma not only inhibit tumors but also participate in tumor metastasis and drug resistance by secreting a variety of cytokines responsible for tumor angiogenesis, immunosuppression, cell growth and other processes (53). It has been reported that tumor-associated macrophages (TAMs) secrete TGF-β (Transforming growth factor-β), which induces tumor cells to undergo EMT and leave the tumor in situ, invade adjacent tissues and enter the circulation, resulting in metastasis. Moreover, tumor cells acquire stem cell-like properties and become resistant to traditional therapies such as chemotherapy, endocrine therapy and radiotherapy in the process of EMT (54). M1 and M2 are the activated macrophage phenotypes with counteracting functions. M1 imparts antitumor immunity whereas M2 favors tumor progression (55). In an interesting observation, macrophages propagated in a conditioned medium from tamoxifen‐sensitive/-resistant breast cancer cells displayed transformation into TAMs (56). In turn, TAMs were found to secrete CCL2 (C-C motif chemokine ligand 2) which further activates the PI3K/AKT/mTOR axis in tumor cells leading to tamoxifen resistance in breast cancer cells (56). In turn, breast cancer cells release TNF-α, activate mTORC1/FOXK1 in TAMs and stimulate M2 polarization with elevated release of CCL2, thereby forming a positive feedback mechanism and offering therapeutic resistance (56). Another study also demonstrated the existence of the positive feedback mechanism between the lactic acid production and HIF-1α/STAT3 axis to promote tamoxifen‐resistant breast cancer cells. The elevated expression of SGLT1 (sodium/glucose cotransporter 1) in tamoxifen-resistant breast cancer cells accelerates glycolysis and subsequent lactic acid production. In turn, lactic acid triggers the HIF-1α/STAT3 axis in M2-like TAMs leading to the release of EGF and subsequent activation of EGFR/PI3K/AKT in breast cancer cells to elevate the expression of SGLT1 and promotion of drug resistance (57). Anastrozole is an aromatase inhibitor used for the treatment of hormone-positive breast cancers. Leptin-dependent signaling cascade has been reported as a contributing factor for the proliferation of aromatase inhibitor-resistant breast cancer cells. Leptin release from anastrozole-resistant breast cancer cells in the TME elevated crosstalk between anastrozole-resistant breast cancer cells and macrophages via CXCR4 (58). Furthermore, TAMs activate the NF-κB/STAT3/ERK signaling pathway in tumor cells by secreting pro-inflammatory cytokines such as TNF-α and IL-6, which are responsible for the phosphorylation and activation of ERα, causing endocrine therapy resistance of breast cancer (59). CD8+ cytotoxic T lymphocytes, a type of tumor infiltrating lymphocytes (TILs), can recognize abnormal cells with foreign antigens and kill them by releasing perforin and granzyme. TAMs can inhibit the antitumor immune response of cytotoxic T lymphocytes. In addition, tumor cells activate immune checkpoints by promoting the binding of programmed cell death ligand 1 (PD-L1) to programmed cell death 1 (PD-1) on T lymphocytes, which inhibits the antitumor immune function of cytotoxic T lymphocytes and causes immune escape of tumor cells (10).

In the process of tumor metastasis, neutrophils and myeloid immunosuppressive cells in the tumor stroma enter the distant organ before the arrival of tumor cells. They not only modify the cellular components and state of the distant organ but also inhibit the immune recognition in the metastatic site and promote the proliferation of tumor cells, which creates a favorable microenvironment for tumor cells to colonize (60). It is generally believed that neutrophils in the TME play a defensive immune role, but in recent years, some studies have found that tumor-associated neutrophils (TANs) exert paracrine effects on the proliferation and differentiation of surrounding tumor cells, resulting in tumor progression (61). Moreover, activated TANs form neutrophil extracellular traps (NETs) in the TME. In NETs, cancer cells exhibit enhanced survival in the circulation and enhanced colonization ability in the new environment, which is conducive to distant metastasis (62). Yang and colleagues found that NETs are not only a “trap for cancer cells”, but also DNA fibers in NETs (NET-DNA) act as chemokines, besides, TANs in distant organs induce tumor cells to metastasize (63). Further studies have shown that the CCDC25 (coiled-coil domain containing protein 25) on the surface of tumor cells binds to NET-DNA as a receptor, which enhances the motility of tumor cells and leads to the occurrence of distant metastasis by activating the integrin-linked kinase (ILK)/β-Parvin signaling pathway.

Tumor cells compete with surrounding immune cells for glucose uptake, limiting the energy supply of TIL cells and leading to the exhaustion of immune cells. In addition, aerobic glycolysis of tumor cells leads to the formation of a microenvironment with hypoxia, high ROS and low pH around the tumor, and the recruitment of myeloid immunosuppressive cells and regulatory T lymphocytes to suppress the function of NK cells and CD8+ cytotoxic T lymphocytes with tumor inhibitory effects, thereby achieving immune escape and tumor progression (10). However, recent studies (64) have found that macrophages rather than tumor cells consume the most glucose in TME, followed by T cells and tumor cells, while tumor cells consume the highest amount of glutamine, which overturns the previous common view of energy metabolism competition in TME and provides a direction for the development of new anticancer therapies.

#### 3.1.3 Tumor stem cell-like cells

In the TME, breast cancer exhibits intratumoral heterogeneity in terms of the existence of various cell types. Breast cancer stem-like cells (BCSCs) are one of the least abundant cell types in the TME with the ability to possess therapeutic resistance and the potential to differentiate into cells of different lineages (8). BCSCs remain dormant and evade the effect of chemotherapeutic agents by expressing drug efflux proteins (65). Importantly, BCSCs remain in the resting phase (G0) and enter the G1 phase of the cell cycle when appropriate stimulus is received. Classical chemotherapeutic agents majorly target actively dividing cells. The dormancy of BCSCs provides them resistance to chemotherapeutic agents and survival benefits over their actively dividing cell counterparts (8). Studies have also found that a small portion of BCSCs travel to distant organs with blood circulation in the early stage and stay dormant. Once they adapt to the new environment, they lose dormancy and enter the cell cycle to differentiate into new tumor cells to achieve metastasis (66). Breast cancer stem cells were found to secrete IL-8 which increased ERα activity, induced tamoxifen resistance and elevated metastatic potential whereas the pharmacological inhibition of IL-8 counteracted tamoxifen resistance with reduction of metastatic potential (67). Mesenchymal stem cells promote tumor metastasis by secreting chemokine ligand 5 (CCL5) and TGF-β, which are responsible for the EMT of tumor cells and the maintenance of stem cell properties (68). The development of therapeutic resistance by BCSC is one of the prime reasons for the failure of endocrine therapy in ER-positive breast cancers (69). Tamoxifen resistant breast cancer cells possess stem-like features (70). The hyperactive PI3K signaling in BCSCs derived from of ERα-positive breast cancer was found to be involved in endocrine resistance (71).

#### 3.1.4 Other cells

Besides the above-mentioned cells, TME also contains other types of cells such as vascular endothelial cells, cancer-associated adipocytes, and stellate cells. Vascular endothelial cells secrete cytokines and ECM components to revive dormant tumor cells, resulting in recurrence or metastasis (72). Peri-tumor adipocytes are modulated and modified by tumor cells to become cancer-associated adipocytes (CAAs), which secrete pro-inflammatory cytokines, growth factors, ECM proteins and metabolites. They regulate the energy metabolism of tumor cells and extracellular matrix remodeling and exert their immune regulatory function, which promotes the occurrence and development of breast cancer (73). CAAs induce the release of fibroblast growth factor (FGF1), which is in charge of the phosphorylation of FGFR in ER-positive breast cancer cells, resulting in resistance to endocrine therapy, furthermore, inhibition of FGFR can reverse the drug-resistant phenotype (74).

### 3.2 Non-cellular components include multiple tangible components and intangible components. They also contribute to endocrine therapy resistance of breast cancer.

#### 3.2.1 ECM

Most of the component proteins of the ECM are produced by stromal cells such as epithelial cells, endothelial cells, and fibroblasts. ECM is made up of proteins, glycoproteins, and proteoglycans (75). Collagen, fibronectin, laminin, and elastin are the major components of ECM (76). ECM is a residence for many growth-regulatory proteins and thereby regulates the functions (such as proliferation, differentiation, and migration) of surrounding cells (77, 78). The structure and integrity of the tissue can be modified by adjusting the spatial arrangement and physical characteristics of the components. The ECM not only participates in tumor initiation and development through its component proteins but also cooperates with soluble factors in the TME to play a role (47). ECM stiffening is a form of ECM remodeling that results from collagen accumulation and collagen fiber aggregation and is closely related to the progression and clinical prognosis of breast cancer (79). Tumor cells regulate the components of the ECM to make it difficult for immune cells and therapeutic drugs to access the tumor cells, thereby achieving immune escape and drug treatment tolerance. The expression of ECM proteins is closely associated with tamoxifen resistance in breast cancer. Analysis of differentially expressed genes in tumor cells from tamoxifen-sensitive and tamoxifen-resistant patients revealed 44 confirmed genes including several ECM proteins such as FN, COL1A1 (collagen type 1 alpha 1 chain), tenascin C, and SPARC (secreted protein acidic and rich in cysteine). Further studies have shown that high expression levels of FN and SPARC are related to early metastasis in breast cancer patients (47). FN in the TME activates the downstream signaling pathway by binding to β1 integrin, causing the phosphorylation of ERα at Ser-118 to regulate the activation of ER, which participates in the process of tamoxifen resistance in breast cancer. Some studies have found that, in the process of ERα activation under the normal culture condition in ER-positive breast cancer cells, estrogen causes the endocytosis of ERα on the cell membrane to form endosomes, some of which enter the nucleus and cause transcriptional effect, and the others are degraded by lysosomes, leading to the gradual reduction of ERα level on the cell membrane. However, in the process of ERα activation under a FN-rich culture condition, estrogen still causes endocytosis of ERα and forms endosomes, part of ERα enters the nucleus and causes transcription effect, while the other part does not enter the lysosome but returns to the cell membrane through FN/β1 integrin signaling pathway, so the expression level of ERα on the cell membrane is not reduced, which attenuates the therapeutic effect of tamoxifen (80). Therefore, they are in support of the notion that the β1 integrin/FN interaction controls the fate of ERα and thus the response to tamoxifen.

Collagen I and IV are also demonstrated to contribute to drug resistance by interacting with integrins present in cancer cells (81, 82). Also, elevated production of collagen reduces the accessibility of cancer cells to chemotherapeutic agents and thereby contributes to therapeutic resistance (83). Jallow and colleagues demonstrated that elevated deposition of collagen I enhanced the density of ECM leading to the resistance against tamoxifen suggesting the importance of targeting ER as well as TME in ER-positive tumors for better clinical outcomes (84). Similarly, laminin was found to trigger tamoxifen resistance in breast cancer cells through α6 integrin. Laminin displayed a protective role in ER-positive breast cancer cells against tamoxifen whereas the pretreatment of these cells with antibodies specific for α6 integrin significantly increased the sensitivity of cells to tamoxifen suggesting the role of laminins in conferring endocrine resistance in TME (85). Overall, these reports suggest that components of ECM play a prominent role in imparting endocrine resistance. Targeting ECM components along with ER may provide significant clinical benefit in endocrine resistant cancers.

#### 3.2.2 Cytokines and growth factors

Cytokines and growth factors are soluble factors in TME that are produced by epithelial cells, epidermal cells, immune cells, and other stromal cells. They are involved in the signal transduction between tumor cells and TME, which affects the growth, differentiation, and movement of tumor cells. In addition, the production of the soluble factors in TME is regulated by tumor cells (42). ER-positive breast cancer cells have been shown to secrete platelet-derived growth factor (PDGF-CC), which binds to platelet-derived growth factor receptor (PDGFR) in CAFs, resulting in increased expression of hepatocyte growth factor (HGF), insulin-like growth factor binding protein 3 (IGFBP3) and stanniocalcin1 (STC1) in charge of endocrine therapy resistance of tumor cells, and targeted inhibition of PDGF-CC restores tumor cell sensitivity to endocrine therapy (86). CAFs and other stromal cells protect tumor cells from the damage of endocrine therapy drugs through a variety of growth factors, proteases and β1 integrin signaling activities. Some studies indicate that immune cells and CAFs secrete IL-6 to activate JAK/STAT3 and PI3K/AKT signaling pathways in tumor cells, which results in the degradation of ERα through the ubiquitin protease pathway, leading to tamoxifen resistance in breast cancer (45).

#### 3.2.3 Others

Tumor-derived exosomes are important tools for communication between tumor cells and TME. They transfer some small RNAs and proteins to recipient cells in the microenvironment for signal transduction. Furthermore, exosomes recruit and modify surrounding stromal cells and induce stromal cell phenotypic transformation, leading to ECM remodeling, tumor angiogenesis, immune tolerance and escape through cytokine, growth factor, chemokine and MMP secretion, contributing to tumor progression (87).

There are many proteases in the extracellular matrix that are involved in the growth and metastasis of tumor cells. MMPs, which are derived from immune cells, endothelial cells, CAFs and hematopoietic progenitor cells in the TME, not only provide a pathway for tumor cell migration by directly degrading the ECM but also regulate the activities of multiple signaling pathways through the lysis of cytokines and growth factors, ultimately leading to increased tumor cell motility (88). Our previous study has found that activation of the ER signaling pathway is significantly reduced in MCF-7 breast cancer cells after tamoxifen resistance. In tamoxifen-resistant cells, estradiol and tamoxifen activate the ER signaling pathway by binding to the GPER, leading to phosphorylation of Src kinase, which in turn activates MMPs on the surface of the cell membrane. The activated MMPs cause the release of heparin-binding epidermal growth factor, which transactivates EGFR and its downstream signaling pathways, including MAPK/ERK and PI3K/AKT signaling pathways, increasing the expression of a series of genes and promoting cell proliferation and migration (89).

The tumor’s local estrogen level is an important factor that affects the effect of endocrine therapy in ER-positive breast cancer, as mentioned above, the expression and activation of aromatase in CAFs are significantly increased, resulting in increased estrogen levels, which is one of the major sources of tumor local estrogen to maintain the growth of tumor cells (41).

## 4 Conclusion and future directions

**Figure 1:**
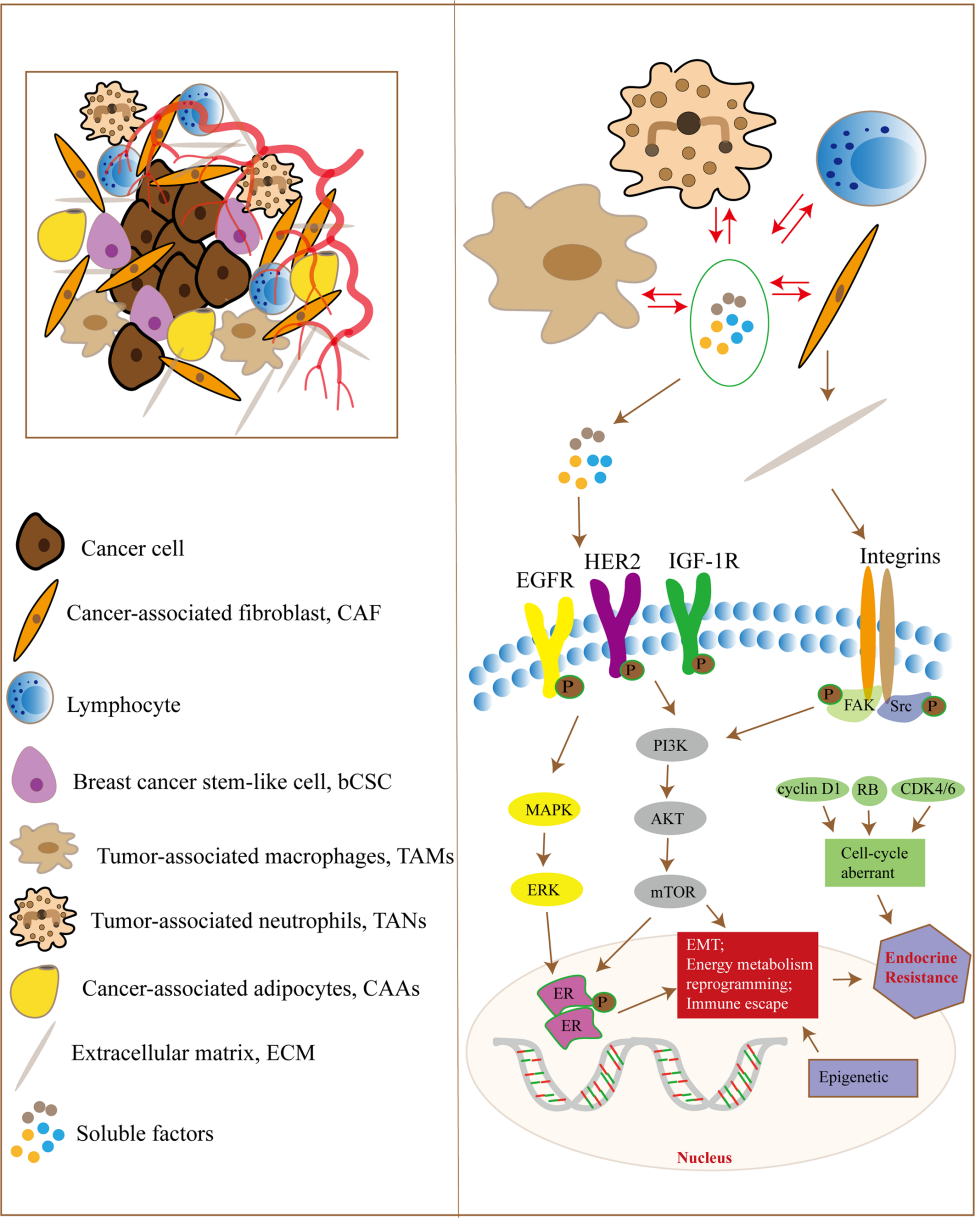
The TME and endocrine therapy resistance in breast cancer. Tumor cells and the TME have complex signaling communication, and the cellular components in the TME interact with each other. They regulate signaling pathways in tumor cells through some soluble factors, ECM proteins, hypoxia and metabolic disorder, which are responsible for EMT, energy metabolism reprogramming and immune escape, leading to endocrine therapy resistance in breast cancer cells. In addition, tumor cells can also regulate the components of the TME through paracrine growth factors and exosomes.

Complex communication exists between tumor cells and stromal cells, as well as between cellular and non-cellular components in the TME, which induces the production of cytokines, growth factors and MMPs involved in EMT, energy metabolism reprogramming, ECM remodeling and immune escape, leading to endocrine therapy resistance of breast cancer (**Figure 1**). Although TME plays an important role in tumor initiation, progression and sensitivity to drug therapy, it should be noted that cancer cells are not objects passively regulated by TME, but actively transform TME to promote cancer cell survival, growth and metastasis. Treatment targeting TME, such as immunotherapy at the early stage of the tumor, may reduce the occurrence of tumor micrometastases and decrease the risk of late recurrence in ER-positive breast cancer patients. In addition, current therapies for hormone receptor-positive advanced breast cancer all target tumor cells, and regulating the interaction between TME and tumor cells may be a promising approach to overcome drug resistance.

## Author contributions

JY: Writing – original draft. LY: Writing – original draft. ZL: Writing – original draft. HZ: Software, Writing – review & editing. QW: Supervision, Writing – review & editing. JH: Writing – review & editing. BW: Writing – original draft, Writing – review & editing. CM: Writing – review & editing, Formal analysis, Validation. GS: Writing – review & editing, Supervision, Formal analysis, Validation, Resources. GW: Supervision, Writing – review & editing.

## Glossary

AR: Androgen receptor
BCSC: Breast cancer stem-like cell
CAA: Cancer-associated adipocyte
CAF: Cancer-associated fibroblast
CCDC25: Coiled-coil domain containing protein 25
CCL2: C-C motif chemokine ligand 2
CDK: Cyclin-dependent kinase
COL1A1: Collagen type 1 alpha 1 chain
COX-2: Cycloxygenase-2
CXCR4: CXC chemokine receptor 4
HDAC: Histone deacetylases
ECM: Extracellular matrix
EGF: Epidermal growth factor
EGFR: Epidermal growth factor receptor
EMT: Epithelial-mesenchymal transition
ER: Estrogen receptor
ESR1: Estrogen receptor alpha gene 1
FGF: Fibroblast growth factor
FGFR: Fibroblast growth factor receptor
FN: Fibronectin
FOXK1: Forkhead box protein K1
GPR30: G-protein-coupled estrogen receptor
HER2: Human epidermal growth factor receptor 2
HGF: Hepatocyte growth factor
HIF: Hypoxia inducible factor
HMGB1: High mobility group box 1
IGF-1R: Insulin-like growth factor-1 receptor
IGFBP3: Insulin-like growth factor binding protein 3
IL-6: Interleukin-6
ILK: Integrin-linked kinase
miRNA: MicroRNA
MMP: Matrix metalloproteinase
mTOR: Mammalian target of rapamycin
mTORC: Mammalian target of rapamycin complex 1
NET: Neutrophil extracellular trap
NF: Normal fibroblast
NF-κB: Nuclear factor-κB
PD-1: Programmed cell death 1
PDGF: Platelet-derived growth factor
PDGFR: Platelet-derived growth factor receptor
PD-L1: Programmed cell death ligand 1
PI3K: Phosphatidylinositol 3-kinase
ROS: Reactive oxygen species
SERD: Selective estrogen receptor degrader
SERM: Selective estrogen receptor modulator
SGLT1: Sodium/glucose cotransporter 1
SPARC: Secreted protein acidic and rich in cysteine
STAT3: Signal transducer and activator of transcription 3
STC1: Stanniocalcin1
TAM: Tumor-associated macrophage
TAN: Tumor-associated neutrophil
TGF-β: Transforming growth factor-β
TIL: Tumor infiltrating lymphocyte
TME: Tumor microenvironment
TNF-α: Tumor necrosis factor-α
VEGFR: Vascular endothelial growth factor receptor
